# Biological plausibility of single-shell NODDI-DTI in non-demented older adults: associations with plasma biomarkers and follow-up cognition

**DOI:** 10.21203/rs.3.rs-9423230/v1

**Published:** 2026-06-05

**Authors:** Giovanni Restuccia, Augusto Ielo, Viviana Lo Buono, Angelo Quartarone, Lilla Bonanno

**Affiliations:** Centro Neurolesi Bonino Pulejo; Centro Neurolesi Bonino Pulejo; Centro Neurolesi Bonino Pulejo; Centro Neurolesi Bonino Pulejo; Centro Neurolesi Bonino Pulejo

**Keywords:** White matter, Diffusion MRI, NODDI-DTI, Plasma biomarkers, pTau181, Neurofilament light chain, Cognitive decline

## Abstract

**Background:**

White matter (WM) degeneration is increasingly recognized as a major feature of aging and neurodegenerative disorders, such as Alzheimer’s disease (AD). NODDI-DTI is a technique that allows the estimation of neurite density index (NDI) and orientation dispersion index (ODI) in white matter from single-shell diffusion tensor imaging. However, it should be interpreted as a tensor-derived approximation and biological support for these metrics is needed. This study aimed to provide multimodal evidence supporting the biological plausibility of NODDI-DTI estimates.

**Methods:**

Demographic, clinical and single-shell DWI data from 42 older adults without dementia were obtained from the Alzheimer’s Disease Neuroimaging Initiative database. We tested associations between NODDI-DTI metrics by performing (i) an exploratory voxel-wise analysis to characterize the spatial distribution and the percentage of statistically significant associations across the white matter (ii) and a tract-based analyses in a priori AD-vulnerable white matter tracts. Finally, we conducted longitudinal analyses to test whether baseline NODDI-DTI metrics and plasma biomarkers were associated with follow-up Montreal Cognitive Assessment (MoCA) performance.

**Results:**

Among the investigated plasma biomarkers, pTau181 was the only biomarker showing widespread significant associations with diffusion metrics, with a substantially more extensive effect for NDI. In the tract-based analyses, higher plasma concentrations of pTau181 were associated mainly with lower NDI in the fornix, inferior fronto-occipital fasciculus and uncinate fasciculus. Higher plasma concentrations of NfL were associated with lower NDI in the fornix, together with opposite ODI effects in the splenium of the corpus callosum and uncinate fasciculus. In the subgroup with follow-up MoCA available, higher baseline ODI in the hippocampal cingulum and higher baseline pTau181 were both independently associated with worse cognitive performance.

**Conclusions:**

These findings suggest that NODDI-DTI may represent a biologically plausible and clinically informative approach to study early WM microstructure, with potential prognostic relevance for subsequent cognitive performance, particularly when full multi-shell acquisitions are not feasible or not available.

## 1 BACKGROUND

White matter (WM) degeneration is increasingly recognized as a major feature of aging and neurodegenerative disorders, such as Alzheimer's Disease (AD) [1, 2]. Alterations in WM microstructure are closely associated with clinical and cognitive outcomes; thus, the discovery and validation of sensitive *in vivo* markers are important goals of neuroscientific research [3–5]. WM microstructure is commonly studied using diffusion-weighted imaging (DWI), a magnetic resonance imaging (MRI) technique that characterizes the random “Brownian motion” of water molecules within brain tissue. Diffusion tensor imaging (DTI) is the most common application of single-shell DWI to assess WM characteristics, and it assumes that the displacement of water molecules follows a Gaussian probability density function [6]. The main indices obtained by fitting a DTI model are fractional anisotropy (FA) and mean diffusivity (MD): while the former measures the directionality of water diffusion on a scale from 0 to 1 (with higher values generally indicating greater WM fiber integrity), the latter measures the overall rate of water diffusion within a voxel. However, DTI has well-recognized limitations, particularly its limited microstructural specificity in regions with non-Gaussian diffusion [6, 7]. Neurite orientation dispersion and density imaging (NODDI) is a biophysical diffusion model developed to address some of these limitations by representing tissue within each voxel as a combination of biologically meaningful compartments [7]. NODDI allows the characterization of brain microstructure by assuming three distinct tissue compartments: intra-neurite space, extra-neurite space and an isotropic free-water compartment (e.g. cerebrospinal fluid [CSF]). The primary NODDI outcome measures are (i) the neurite density index (NDI), which reflects the tissue signal attributed to restricted diffusion in the neurites, (ii) the orientation dispersion index (ODI), which measures the organization of the neurites by estimating their dispersion, and (iii) the isotropic volume fraction (ISO), indexing the contribution of free water in each voxel [7]. However, the application of this technique typically requires multi-shell DWI acquisitions that sample the signal at multiple b-values (e.g. b = 0, 1000, 2000 s/mm^2^) [7]. These acquisitions remain less common in routine clinical practice and are often unavailable in legacy datasets, limiting broad clinical feasibility and retrospective validation. To address this limitation, the NODDI-DTI model simplifies the original NODDI framework, allowing the estimation of NDI and ODI in WM by using FA and MD from single-shell DTI [8, 9]. In parallel, plasma biomarkers, quantified via ultra-sensitive assays, such as Single Molecule Array Technology (SIMOA), provide a reliable and minimally invasive approach to detect early neuropathological and neurodegenerative changes even in cognitively normal subjects [4, 10, 11]. Biomarkers such as the beta-amyloid (Aβ) 42/40 ratio, neurofilament light chain (NfL), phosphorylated tau species (e.g. pTau181 and pTau217) and glial fibrillary acidic protein (GFAP) reflect key brain processes such as amyloid plaque deposition, axonal damage and tau-related pathology, providing valuable insights into cognitive decline [11–14]. Prior multimodal evidence suggests that these biomarkers are associated with diffusion-derived microstructural measures. For instance, higher NfL is generally associated with lower WM NDI and higher or lower ODI [10, 15, 16]; tau pathology is consistently linked to reduced NDI [5, 15, 16], whereas direction and magnitude of associations with ODI appear more heterogeneous [5, 16].

However, NDI and ODI estimated from single-shell NODDI-DTI should be interpreted as tensor-derived approximations that rely on specific assumptions in WM, hence biological support for these metrics is needed [8]. To our knowledge, evidence directly examining the associations between blood or CSF biomarkers and NODDI-DTI metrics remains limited. To address this gap, we performed (i) an exploratory skeleton-based voxel-wise analysis to characterize the spatial distribution and the percentage of statistically significant associations in WM voxels, and (ii) a tract-based analysis focusing on *a priori* AD-vulnerable WM tracts. Finally, we assessed whether baseline NODDI-DTI metrics and plasma biomarkers were associated with future global cognitive performance. By integrating diffusion-derived WM microstructure metrics, plasma biomarkers of neurodegeneration and AD-related pathology and longitudinal cognitive outcomes, this study aimed to provide multimodal evidence supporting the biological plausibility and clinical relevance of NODDI-DTI estimates.

## 2 METHODS

### 2.1 Participants

Demographic, clinical and single-shell DWI data from 42 participants were obtained from the Alzheimer’s Disease Neuroimaging Initiative (ADNI) database (https://ida.loni.usc.edu/). Selection criteria were defined *a priori* on the basis of data availability and protocol harmonization rather than clinical diagnosis. Specifically, participants were required to have the same single-shell DWI acquisition protocol, an available plasma biomarker panel measured using the Quanterix SIMOA ^®^ platform and a baseline Montreal Cognitive Assessment (MoCA) score. A subgroup of participants (*n* = 25) also had MoCA data available at the latest follow-up visit. Given the restrictive eligibility criteria, the absence of dementia, and the overall preservation of global cognition at baseline, participants were analyzed as a single cohort of older adults with largely preserved cognition rather than being stratified by clinical diagnosis. This approach was supported by baseline Clinical Dementia Rating (CDR) global scores (ranging from 0 to 0.5) and by a mean Mini Mental State Examination (MMSE) score above the conventional clinical cut-off (≥ 24).

### 2.2 DWI acquisition and plasma biomarkers

DWI data were acquired on a 3.0 Tesla MRI scanner (Discovery MR750, GE Healthcare) using a 2D axial single-shot spin-echo echo-planar imaging DTI sequence with b = 1000 s/mm^2^, 48 diffusion-encoding directions, and 6 b0 volumes. Additional acquisition parameters were as follows: repetition time (TR) = 7800 ms, minimum echo time (TE), field of view (FOV) = 23.2 cm, matrix size = 116 × 116, slice thickness = 2.0 mm with no interslice gap, and right–to-left phase-encoding direction. Data were acquired using an 8-channel high-resolution brain array coil. Plasma biomarkers were quantified on the Quanterix SIMOA ^®^ HD-X platform using the HDX-214 assay for Aβ40, Aβ42, GFAP, NfL, the pTau181 Advantage 2.1 assay (HDX-230) and pTau217 v2 assay (HDX-123). Biomarker concentrations (pg/mL) were retrieved from the ADNI repository and log-transformed before statistical analysis to reduce skewness. For amyloid measures, the Aβ42/40 ratio was additionally calculated.

### 2.3 Image processing

Raw diffusion data were visually inspected for quality and converted from DICOM images to NIfTI format using *dcm2niix* [17]. Preprocessing included denoising and Gibbs ringing removal using MRtrix3 (version 3.0) [18], followed by correction for eddy-current distortions and subject motion using the FMRIB Software Library (FSL, version 6.0.5.2) [19]. Preprocessed outputs were visually inspected for each subject to verify the adequacy of motion and eddy-current correction. Brain extraction was performed on the first b0 volume using BET in order to generate a subject-specific brain mask. This mask was used both as input to eddy and to constrain subsequent model fitting to brain tissue. The diffusion tensor was then fitted voxel-wise using diffusion imaging in Python (DIPY, version 1.11.0), from which FA and MD maps were derived. NDI and ODI maps were subsequently estimated using the NODDI-DTI framework [8], a single-shell approximation of the original NODDI model [7], implemented through a custom Python script based on the procedure described by Edwards et al. [8]. Using FSL tract-based spatial statistics (TBSS), FA maps were non-linearly registered to the *FMRIB58_FA* template in MNI152 standard space. A mean FA image was then generated across all participants and thresholded at 0.2 to create the mean WM skeleton, thereby reducing gray matter contamination and partial volume effects. Individual FA maps were projected onto the skeleton and the quality of the resulting registrations and skeleton projections was visually checked. The same warps and projection parameters were then applied to NDI and ODI maps using the TBSS non-FA pipeline.

### 2.4 Statistical analysis

Statistical analyses were designed to examine the associations between FA and NODDI-DTI metrics (NDI and ODI) and plasma biomarkers (Aβ42/40 ratio, NfL, GFAP, pTau181, and pTau217). We first performed voxel-wise linear modeling using TBSS to test associations across the WM skeleton. This approach allowed us to map the spatial distribution of significant effects along the WM skeleton and to quantify them as the percentage of skeleton voxels surviving correction for multiple comparisons. This descriptive metric was used to provide an intuitive estimate of the spatial extent of significant associations across the WM skeleton. Because several variables showed non-normal distributions according to the Shapiro–Wilk test and given the relatively small sample size, associations were assessed using partial Spearman’s rank correlations, which provide a robust non-parametric estimate of monotonic relationships between variables. In addition, we conducted a tract-based ROI analysis in a priori selected AD-vulnerable WM tracts by extracting mean values for each diffusion metric and correlating them with each plasma biomarker. This approach was carried out to enhance anatomical and clinical interpretability and to improve statistical power. Lastly, we tested longitudinal associations between baseline NODDI-DTI metrics in the selected WM tracts and MoCA performance at follow-up. All statistical analyses, except for the voxel-wise TBSS analyses, were performed in JASP (version 0.95.4.0, https://jasp-stats.org/). To account for multiple testing in ROI and correlation analyses, *p*-values were adjusted using the false discovery rate (FDR) procedure according to the Benjamini–Hochberg method. Details of each analysis are reported below.

#### Voxel-wise analysis

We used TBSS’s *randomise* function [20] to test associations between skeletonized FA, NDI and ODI maps and plasma biomarkers in separate models adjusted for age and sex. The following general linear model (GLM) was fitted to each voxel for each diffusion metric:

FA/NDI/ODI=β0+β1*plasmabiomarker+β2*age+β3*sex

*Randomise* was run with 5000 permutations and threshold-free cluster enhancement and family-wise error correction (TFCE-FWE) to correct for multiple comparisons across voxels (*p* < 0.05). We then created binary masks including only voxels exhibiting significant associations between diffusion metrics and plasma biomarkers (*p* < 0.05) and calculated their percentage through the *fslstats* module. Finally, the significant voxels were highlighted with *tbss_fill* and displayed on the 1-mm MNI152 T1 template.

#### Tract-wise ROI analyses

FA, NDI and ODI metrics were extracted for each participant using the *JHU ICBM-DTI-81* WM atlas within *a priori* AD-vulnerable pathways. ROIs included: splenium of the corpus callosum (SCC), fornix (FX), cingulate gyrus cingulum (CGC), hippocampal cingulum (CH), inferior fronto-occipital fasciculus (IFOF), and uncinate fasciculus (UF). Binary masks for each tract were applied to the individual normalized, skeletonized maps and mean values were extracted and bilaterally averaged. To characterize the strength and direction of the associations between conventional DTI and NODDI-DTI metrics within the selected WM pathways, we conducted within-tract partial Spearman’s correlations between FA and NDI, and between FA and ODI, controlling for age and sex. We then examined cross-sectional associations between FA, NDI and ODI tract metrics and log-transformed plasma biomarkers with partial Spearman’s rank correlations adjusted for age, sex and education. The resulting significant associations between NODDI-DTI metrics and plasma biomarkers were subsequently tested using multiple regression models, with the diffusion metric as the dependent variable and plasma biomarker as the independent variable while accounting for age, sex and education as covariates.

#### Associations with global cognitive performance

We first performed cross-sectional partial Spearman’s correlations adjusted for age, sex, and education in the whole sample (*n* = 42) between global cognition (MoCA) and NODDI-DTI metrics in the *a priori* AD-vulnerable WM tracts in the whole cohort. Subsequently, we identified a subset of participants (*n* = 25) who also had MoCA available at the latest follow-up (mean follow-up time: 4.8 years) and conducted partial correlations between baseline NODDI-DTI in WM tracts and MoCA score at follow-up while controlling for age, sex, education, and baseline MoCA. In parallel, the same correlation analyses were also performed between baseline plasma biomarkers and MoCA score at follow-up. Variables showing significant associations in the correlation analyses were subsequently entered into a hierarchical multiple linear regression analysis to evaluate the incremental value of significant variables in predicting follow-up MoCA score. Covariates (age, sex, education, baseline MoCA, and follow-up time) were entered in the first block (M0) whereas variables showing significant associations were added in subsequent nested blocks. Changes in explained variance (ΔR^2^) and the corresponding *p*-values for model improvement were reported at each step. For each predictor, unstandardized (B) and standardized regression coefficients (β), 95% confidence intervals (CIs), and *p*-values were reported. Multicollinearity among predictors was assessed using variance inflation factors (VIFs), with values < 3 indicating no relevant collinearity. Model diagnostics also included inspection of residuals, leverage, and Cook’s distance.

## 3 RESULTS

### 3.1 Demographic and clinical characteristics of the participants

Demographic and clinical characteristics are reported in Table 1. Participants (*n* = 42) were older adults (mean age 79.09 ± 7.13 years) with a relatively high education level (16.48 ± 3.02 years) and a balanced sex distribution (24 females and 18 males). Global cognition at baseline was preserved, with a mean MMSE score of 28.02 ± 3.47 and a mean MoCA score of 24.83 ± 4.99. Baseline CDR-global score was low in the overall sample (0.13 ± 0.22), consistent with minimal clinical impairment. Plasma biomarker concentrations at baseline were as follows (mean ± SD): pTau181 21.06 ± 11.29 pg/mL, pTau217 0.39 ± 0.34 pg/mL, NfL 24.28 ± 11.90 pg/mL, and GFAP 198.20 ± 89.71 pg/mL. Values were broadly similar in the follow-up subgroup (pTau181 19.62 ± 11.59, pTau217 0.32 ± 0.26, NfL 25.26 ± 14.00, GFAP 207.90 ± 99.33). A subset of participants (*n* = 25) also had follow-up MoCA data available; this subgroup was comparable in age (78.16 ± 7.20 years), education (15.76 ± 3.24 years) and sex distribution (16/9) and showed a baseline MoCA of 25.40 ± 4.53 with a mean follow-up MoCA of 22.76 ± 5.54. The mean follow-up time was 4.8 years.

### 3.2 Results of the voxel-wise analysis

**Figure 1:**
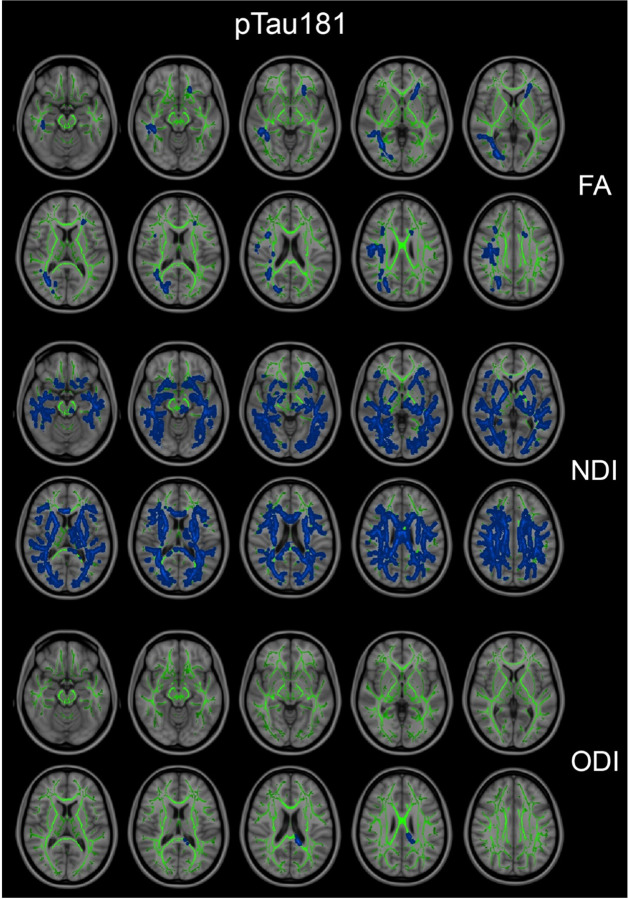
Significant WM voxels showing negative associations (blue) between pTau181 and FA, NDI and ODI displayed on the 1-mm MNI152 T1 template. Significant voxels were highlighted using tbss_fill on the mean FA skeleton (green) which was derived from the average FA maps across participants (TFCE-FWE, p < 0.05 adjusted for age and sex).

Among the investigated plasma biomarkers, pTau181 was the only biomarker showing widespread significant associations with diffusion metrics (Figure 1). In particular, significant negative associations were observed in 2.8% of the WM skeleton voxels for FA, 29.0% for NDI and 0.09% for ODI.

### 3.3 Results of the tract-based analysis

Within-tract correlations showed a positive coupling between FA and NDI across all selected AD-vulnerable tracts (Table 2), indicating substantial shared variance between these indices. In contrast, FA–ODI relationships were tract-dependent: significant inverse associations emerged in the SCC (ρ = −0.93, *p* < 0.001), FX (ρ = −0.44, *p* = 0.013), CGC (ρ = −0.40, *p* = 0.024), and CH (ρ = −0.39, *p* = 0.031), whereas no significant associations were observed in the IFOF or UF.

**Figure 2:**
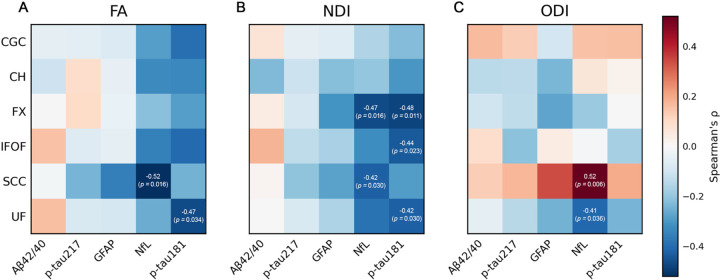
Heatmap representation of Spearman's correlation coefficients between diffusion metrics in selected WM tracts and plasma biomarkers adjusted for age, sex, and education. Numeric correlation coefficients and FDR corrected p-values are shown only for significant associations (p < 0.05). Panels display correlations for FA (**A**), NDI (**B**) and ODI (**C**). The color scale represents the strength and direction of the correlations, with blue indicating negative associations and red indicating positive associations. **Abbreviations**: CGC = Cingulum (Cingulate Gyrus); CH = Cingulum (Hippocampus); FX = Fornix; IFOF = Inferior fronto-occipital fasciculus; SCC = splenium of corpus callosum; UF = Uncinate Fasciculus.

**Figure 3:**
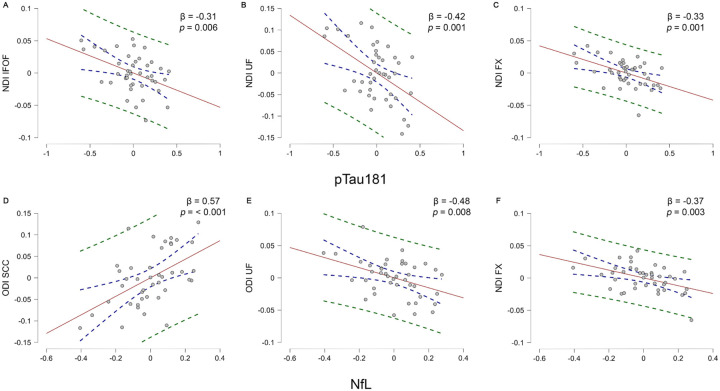
Partial regression plots illustrating significant associations between plasma biomarkers and NODDI-DTI metrics after adjustment for age, sex, and education. Both predictor and outcome values are shown as residualized values from the corresponding multiple linear regression models. Higher plasma pTau181levels were associated with lower NDIin the inferior fronto-occipital fasciculus (IFOF, **A**), uncinate fasciculus (UF, **B**), and fornix (FX, **C**). Higher plasma NfL levels were associated with higher ODI in the splenium of the corpus callosum (SCC, **D**), lower ODI in the UF (**E**), and lower NDI in the FX (**F**). The solid red line represents the fitted regression line, whereas the dashed blue and green lines indicate the 95% confidence interval and the 95% prediction interval respectively. Standardized regression coefficients (β) and *p* values are reported in each panel.

The heatmap in Fig. 2 shows significant partial correlations between diffusion metrics and plasma biomarkers. Associations with NODDI-DTI that were significant in the partial correlation analyses were further analyzed with multiple linear regression analyses (Fig. 3). Higher pTau181 was associated with lower NDI in the FX $\left(p=0.001,\beta =-0.33,R_{\text{adj}}^{2}=0.68\right)$, IFOF $\left(p=0.006,\beta =-0.31,R_{\text{adj}}^{2}=0.60\right)$ and UF $\left(p=0.001,\beta =-0.42,R_{\text{adj}}^{2}=0.49\right)$. Higher NfL was associated with lower NDI in the FX $\left(p=0.003,\beta =-0.37,R_{\text{adj}}^{2}=0.67\right)$, lower ODI in the UF $\left(p=0.008,\beta =-0.48,R_{\text{adj}}^{2}=0.26\right)$ and with higher ODI in the SCC $\left(p=<0.001,\beta =0.57,R_{\text{adj}}^{2}=0.42\right)$.

### 3.4 Results of associations with MoCA score

**Figure 4:**
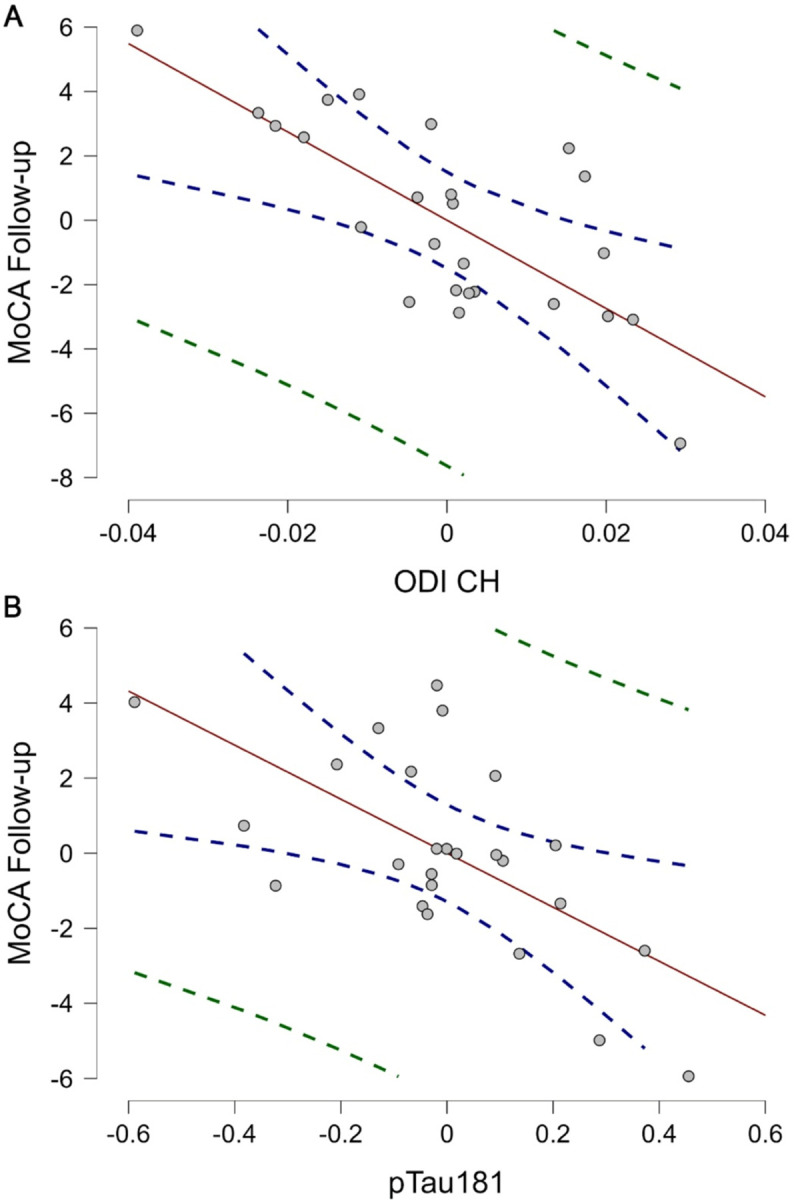
Partial regression plots showing the independent association of baseline ODI in the hippocampal cingulum (CH, **A**) and baseline plasma pTau181 (**B**) with follow-up MoCA score. Axes represent residualized values after accounting for the other covariates included in the model. In both panels, higher baseline values were associated with lower cognitive performance at follow-up. The solid red line represents the fitted regression line, whereas the dashed blue and green lines indicate the 95% confidence interval and the 95% prediction interval respectively.

In the whole study cohort, no significant cross-sectional correlations were found between MoCA score and NODDI-DTI metrics in WM tracts. In the subgroup (*n* = 25) with follow-up MoCA available (mean follow-up time, 4.8 ± 1.2 years), higher baseline ODI in the CH (ρ = −0.50, *p* = 0.008) and higher baseline pTau181 (ρ = −0.57, *p* = 0.008) were associated with lower follow-up MoCA performance. In the hierarchical regression analysis (Table 3), the base model (M0), including age, sex, education, baseline MoCA and follow-up time, explained 58.9% of the variance in follow-up MoCA score $\left(R^{2}=0.589;R_{\text{adj}}^{2}=0.481,p=0.003\right)$. Adding baseline plasma pTau181 significantly improved the model (M1), explaining an additional 12.0% of the variance (ΔR^2^ = 0.120, *p* = 0.014). Finally, inclusion of baseline ODI in the CH further improved the model (M2), accounting for an additional 16.1% of the variance (ΔR^2^ = 0.161, *p* < 0.001), with the final model explaining 87.1% of the variance in follow-up MoCA score $\left(R^{2}=0.871;R_{\text{adj}}^{2}=0.818,p<0.001\right)$. Baseline ODI in the CH (β = −0.449, *p* < 0.001) and baseline plasma pTau181 (β = −0.344, *p* = 0.004) were independently associated with follow-up MoCA (Table 4; Fig. 4).

## 4 DISCUSSION

In this study, we investigated whether single-shell NODDI-DTI metrics showed biologically meaningful associations with plasma biomarkers of neurodegeneration and AD-related pathology, and whether they were associated with future cognitive outcomes, by combining exploratory voxel-wise and tract-based analyses. NODDI-DTI is an approximation framework designed to compute NDI and ODI metrics in WM tracts from tensor-derived maps (i.e. FA and MD). The main difference between this and the original NODDI framework is that the latter requires data from multi-shell DWI acquisitions, which are not typically implemented in routine clinical practice. In addition, multi-shell imaging is often not available in legacy datasets. In this context, the use of single-shell NODDI-DTI may enhance clinical feasibility and allow retrospective validation in datasets in which multi-shell data are not provided.

The voxel-wise analysis provided the clearest signal of the study: pTau181 was the only plasma biomarker associated with widespread WM alterations, with an effect that was substantially more extensive for NDI than for FA or ODI. Specifically, significant negative associations involved 29% of the white matter skeleton for NDI, compared with 2.8% for FA and only 0.09% for ODI. This pattern suggests that these metrics are not interchangeable and may differ in their sensitivity to tau-related microstructural damage. A plausible interpretation is that pTau181-related WM damage is captured by both indices but emerges more extensively with NDI because it may reflect the axonal density component, whereas FA reflects a less specific signal influenced by multiple microstructural features. Prior experimental work has linked pTau181 signals to axons with damaged myelin sheaths in a mouse model [21], supporting a relationship between tau pathology and WM injury. In humans, higher plasma pTau181 concentrations have been associated with greater WM lesion burden [22] and with lower FA and higher diffusivity across Alzheimer’s disease stages [23]. Furthermore, greater cortical tau-PET burden was associated not only with decreased FA and increased diffusivity, but also with reduced NDI, whereas ODI changes were much less prominent, suggesting that tau-related WM damage may be more closely linked to axonal packing loss than to dispersion alone [2]. Consistent with these results, Parker and colleagues [5] also showed that tau-PET in the entorhinal cortex was strongly associated with NDI in the CH and UF and with ODI in the FX; by contrast, they found no associations with FA and MD in the same WM tracts. In this context, the much broader voxel-wise signal observed for NDI than for FA may reflect a greater sensitivity of NDI to the neurite-related component of tau-associated WM damage. At the same time, the near absence of an ODI effect suggests that the voxel-wise pTau181 effect was not driven by a generic increase in all tensor-derived metrics.

To better characterize the magnitude and direction of the associations between conventional tensor metrics and NODDI-DTI metrics, we performed partial correlations between FA and NDI/ODI in selected WM pathways. Robust positive correlations between FA and NDI emerged in all the analyzed tracts, although with variable magnitude, suggesting substantial shared variance between these two indices. By contrast, correlations between FA and ODI were more tract-dependent. Specifically, we found significant inverse correlations in the SCC, FX, CH and CGC but not in the IFOF or UF. More specifically, very high coefficients were observed only in selected bundles, particularly in the SCC for both FA–NDI and FA–ODI, and in the UF and IFOF for FA–NDI. In the single-shell NODDI-DTI framework, NDI and ODI are derived from tensor maps with an algorithmic approximation, therefore, some degree of shared variance is expected [8]. However, the fact that very strong correlations were confined to specific tracts suggests that the overlap between these metrics is not uniform across WM pathways. Rather than indicating simple redundancy, this pattern may reflect tract-dependent differences based on how well tensor-derived approximations capture the underlying structural features, particularly given that NODDI-DTI relies on assumptions that are expected to hold best in relatively coherent WM with limited CSF contamination. These findings support the idea that while NDI may partially overlap with FA in some tracts, ODI may index different, non-redundant information related to axonal dispersion or tract geometry.

The tract-based analyses add anatomical specificity to the voxel-wise findings: pTau181 and NfL showed distinct but complementary profiles. Higher plasma concentrations of pTau181 were associated mainly with lower NDI in the FX, IFOF, and UF. Consistent with our findings, prior ADNI evidence showed that higher plasma pTau181 levels are associated with lower FA and higher diffusivity across multiple WM regions, including limbic pathways such as the CH and FX, suggesting that blood pTau181 reflects microstructural WM vulnerability across AD progression [23]. In addition, although Parker et al. [5] investigated tau-PET rather than plasma pTau181, their findings provide important biological support for the anatomical relevance of our tract-wise results, as entorhinal tau was associated with NDI in the CH and UF, whereas FA showed no significant tau-related associations. More recent studies have also reported an association between pTau181 and medial temporal lobe gray matter, with higher plasma concentrations associated with greater atrophy, in line with the topography of the WM tracts identified here [11, 24]. By contrast, although voxel-wise analysis did not show any significant correlation between NODDI-DTI and NfL, in the tract-based analysis higher plasma concentrations of NfL were associated with lower NDI in the FX, together with opposite ODI effects in the SCC and UF. This pattern is consistent with prior ADNI evidence showing that higher plasma NfL levels were associated with lower FA in the FX, callosal regions and associative pathways such as the IFOF and UF [25]. In addition, recent work using an advanced modified version of NODDI showed that lower NDI values were associated with higher plasma NfL concentrations across multiple WM regions, although no associations with ODI were reported [26]. This work supports the interpretation of NDI as an imaging correlate of axonal integrity and NfL as a biomarker of axonal degeneration (Khalil et al., 2024). Furthermore, these results suggest that, even as a tensor-derived single-shell approximation, NODDI-DTI may add interpretative value beyond conventional DTI by separating neurite density-related and orientation dispersion-related features [7, 8].

A prediction model combining NODDI-DTI metrics from selected WM tracts and plasma biomarker concentrations was used to investigate whether their multimodal integration was associated with future cognitive outcomes. In a subgroup of 25 participants with MoCA available at follow-up, higher baseline ODI in the CH and higher baseline pTau181 were both independently associated with worse cognitive performance. These findings go beyond the cross-sectional associations, suggesting that tract-specific microstructural markers and blood tau markers may provide important prognostic information. Notably, the CH is a particularly plausible tract in this context because it is fundamental to the episodic memory network and strongly implicated in early AD-related neurodegeneration [3, 5, 27–29]. Indeed, in two recent studies [5, 28], diffusion microstructural alterations in medial temporal WM, including the CH, were associated with memory performance and AD pathology. In parallel, longitudinal population-based studies indicate that combining WM injury metrics with plasma AD biomarkers improves the prediction of cognitive decline and dementia risk over time [4, 5]. Our data extend this literature by showing that even in a small and clinically preserved cohort, baseline microstructural disorganization in the CH and baseline plasma pTau181 retained independent explanatory value for future global cognition. Finally, no significant cross-sectional associations were found between MoCA and NODDI-DTI measures in the full cohort. This may not be surprising, since in relatively preserved older adults, microstructural alterations may precede measurable deficits on neurocognitive tests, and tract-specific diffusion abnormalities may become prognostically informative before they are strongly reflected in concurrent MoCA performance [1, 3, 28].

No significant associations for plasma Aβ42/40 and GFAP were observed. There is growing evidence in the diffusion MRI literature that both plasma Aβ42/40 and GFAP can relate to WM microstructure [12, 13, 30–32]; however, these associations appear to be subtler and more context-dependent than those observed for pTau181, often emerging in amyloid-enriched groups, in larger longitudinal or cognitively impaired cohorts. In this context, rather than suggesting biological irrelevance or the absence of an underlying relationship, the lack of associations with both DTI and NODDI-DTI metrics in our study may reflect limited biological enrichment, restricted cognitive variability and weak statistical power.

Regarding pTau217, the null findings should be considered in light of the still limited and inconclusive literature linking this biomarker to diffusion WM microstructure metrics, despite its growing recognition as a highly accurate indicator of amyloid positivity and early disease progression [33, 34]. Accordingly, the absence of significant associations in our study should be interpreted cautiously and may reflect sample characteristics and limited power rather than the absence of an underlying biological relationship.

### Limitations and future perspectives

Although the findings support the biological plausibility of NODDI-DTI, several limitations must be considered. First, the sample size was modest, especially for the longitudinal subgroup, which limits the stability of effect estimates, reduces power to detect weaker associations and may increase the risk of overfitting and coefficient instability in the longitudinal regression model. Second, the cohort was selected on the basis of MRI protocol harmonization and SIMOA biomarker availability rather than biological stratification, and participants were treated as a single largely cognitively preserved group of older adults; while methodologically pragmatic, this likely reduced heterogeneity in both biomarkers and cognition. Third, even though this was not an objective of the study, no independent amyloid or tau classification based on PET or CSF was available, limiting the possibility of positioning the findings along the AD continuum and of interpreting null associations in biologically defined subgroups. Finally, plasma biomarkers were available at a single time point, and the longitudinal component was restricted to MoCA follow-up, precluding the assessment of parallel changes over time in blood biomarkers, WM microstructure and cognition. Future studies in larger and biologically better characterized cohorts are needed to replicate these findings and to determine whether NODDI-DTI metrics retain their associations with plasma biomarkers across different stages of the AD continuum. Longitudinal multimodal designs including repeated plasma biomarker assessments, cognitive follow-up, independent amyloid/tau classification, and direct comparison with multi-shell NODDI measures will be important to clarify the robustness, biological specificity, and potential prognostic relevance of these single-shell diffusion-derived metrics.

## 5 CONCLUSIONS

Overall, our findings support the biological plausibility and potential clinical relevance of NODDI-DTI, an approximation of NDI and ODI metrics derived from single-shell standard DTI acquisitions, as a potentially sensitive marker of WM microstructural vulnerability in aging individuals at risk of AD-related neurodegenerative change. In particular, we demonstrated convergent associations between NODDI-DTI metrics and plasma biomarkers, especially pTau181, suggesting that these single-shell diffusion-derived metrics may capture biologically relevant microstructural variation. This convergence was observed in two complementary ways: first, through a skeleton-wide association between pTau181 and NODDI-DTI metrics; second, through a tract-specific pattern of associations with NfL and pTau181 involving the FX, UF, IFOF and CH, which supports the interpretation that NODDI-DTI may be able to detect microstructural changes in AD-vulnerable WM tracts. Further, the independent contribution of baseline CH ODI and plasma pTau181 to follow-up MoCA also indicates that these markers are not only biologically informative but may carry prognostic value for subsequent cognitive performance. Within this framework, plasma pTau181 emerged as the plasma biomarker most consistently linked to NODDI-DTI measures of microstructure vulnerability and to later cognitive performance. Taken together, these results suggest that NODDI-DTI may represent a biologically plausible and clinically informative approach to study early WM microstructure, with potential prognostic relevance for subsequent cognitive performance, particularly when full multi-shell acquisitions are not feasible or not available.

## Figures and Tables

**Table 1:** Demographic and clinical characteristics of the study cohort at baseline and follow-up.

*Demographics*	All subjects (*n* = 42)	Subjects with FU (*n* = 25)
Age at BL (years)	79.09 ± 7.13	78.16 ± 7.20
Education at BL (years)	16.48 ± 3.02	15.76 ± 3.24
Gender (F/M)	24/18	16/9
*Neuropsychological outcomes*		
MMSE at BL (score)	28.02 ± 3.47	-
MoCA at BL (score)	24.83 ± 4.99	25.40 ± 4.53
MoCA at FU (score)	-	22.76 ± 5.54
CDR-global at BL (score)	0.13 ± 0.22	
*Plasma biomarkers*		
pTau181 at BL (pg/mL)	21.06 ± 11.29	19.62 ± 11.59
pTau217 at BL (pg/mL)	0.39 ± 0.34	0.32 ± 0.26
NfL at BL (pg/mL)	24.28 ± 11.90	25.26 ± 14.00
GFAP at BL (pg/mL)	198.20 ± 89.71	207.90 ± 99.33

Continuous variables are presented as mean ± standard deviation, categorical variables are presented as counts.

**Abbreviations**: BL = baseline; CDR = Clinical Dementia Rating Scale; FU = follow-up; GFAP = Glial Acidic Fibrillary Protein; MMSE = Mini Mental State Examination; MoCA = Montreal Cognitive Assessment; NfL = Neurofilament Light Chain; ptau181 = phosphorylated tau 181; ptau217 = phosphorylated tau 217.

**Table 2:** Within-tract correlations between FA and NODDI-DTI metrics across selected WM tracts

Tract	FA vs. NDI	FA vs. ODI
SCC	ρ = 0.82, *p* < 0.001	ρ = −0.93, *p* < 0.001
FX	ρ = 0.50, *p* = 0.004	ρ = −0.44, *p* = 0.013
CGC	ρ = 0.44, *p* = 0.012	ρ = −0.40, *p* = 0.024
CH	ρ = 0.54, *p* = 0.002	ρ = −0.39, *p* = 0.031
IFOF	ρ = 0.69, *p* < 0.001	ns
UF	ρ = 0.75, *p* < 0.001	ns

Cells report Spearman’s ρ and FDR adjusted p-value (p). Correlations were adjusted for age and sex.

**Abbreviations**: CGC = Cingulum (Cingulate Gyrus); CH = Cingulum (Hippocampus); FX = Fornix; IFOF = Inferior fronto-occipital fasciculus; ns = not significant; SCC = splenium of the corpus callosum; UF = Uncinate Fasciculus

**Table 3:** Hierarchical nested regression models predicting follow-up MoCA score.

Model	Predictors included	R^2^	Adjusted R^2^	ΔR^2^	*p* (ΔR^2^)	*p* model
M0	Age, Sex, Education, MoCA at BL, FU (years)	0.589	0.481	-	0.003	0.003
M1	M0 + pTau181 at BL	0.710	0.613	0.120	0.014	< 0.001
M2	M1 + ODI CH at BL	0.871	0.818	0.161	< 0.001	< 0.001

**Abbreviations**: BL = baseline; CH = Cingulum (Hippocampus); FU = follow-up time; MoCA = Montreal Cognitive Assessment.

**Table 4:** Predictors included in the final hierarchical regression model.

Predictors	B	β	*p*	95% CI for B
Age	0.077	0.101	0.319	−0.082 to 0.236
Sex	0.151	0.013	0.894	−2.202 to 2.504
Education	0.502	0.294	0.007	0.154 to 0.849
MoCA at BL	0.809	0.661	< 0.001	0.494 to 1.124
FU (years)	−0.472	−0.104	0.356	−1.522 to 0.577
pTau181 at BL	−7.196	−0.344	0.004	−11.678 to −2.713
ODI CH at BL	−137.167	−0.449	< 0.001	−199.965 to −74.369

**Abbreviations**: BL = baseline; CI = confidence interval; CH = Cingulum (Hippocampus); FU = follow-up time; MoCA = Montreal Cognitive Assessment.

## Data Availability

The datasets analysed during the current study are available from the Alzheimer’s Disease Neuroimaging Initiative (ADNI) repository at http://www.adni-info.org.

## ABBREVIATIONS

Aβ: Beta-amyloid
AD: Alzheimer’s disease
ADNI: Alzheimer’s Disease Neuroimaging Initiative
CGC: Cingulate gyrus cingulum
CH: Hippocampal cingulum
CDR: Clinical Dementia Rating
CI: Confidence interval
CSF: Cerebrospinal fluid
DICOM: Digital Imaging and Communications in Medicine
DIPY: Diffusion Imaging in Python
DTI: Diffusion tensor imaging
DWI: Diffusion-weighted imaging
FA: Fractional anisotropy
FDR: False discovery rate
FOV: Field of view
FSL: FMRIB Software Library
FX: Fornix
FU: Follow-up
GFAP: Glial fibrillary acidic protein
GLM: General linear model
IFOF: Inferior fronto-occipital fasciculus
ISO: Isotropic volume fraction
MD: Mean diffusivity
MMSE: Mini Mental State Examination
MoCA: Montreal Cognitive Assessment
MRI: Magnetic resonance imaging
NDI: Neurite density index
NfL: Neurofilament light chain
NODDI: Neurite orientation dispersion and density imaging
ODI: Orientation dispersion index
PET: Positron emission tomography
ROI: Region of interest
SCC: Splenium of the corpus callosum
SIMOA: Single Molecule Array
TBSS: Tract-Based Spatial Statistics
TE: Echo time
TFCE-FWE: Threshold-free cluster enhancement with family-wise error correction
TR: Repetition time
UF: Uncinate fasciculus
VIF: Variance inflation factor
WM: White matter
